# Supplementary material for: Public preferences for delayed or immediate antibiotic prescriptions in UK primary care: A choice experiment
Source: PLoS Med. 2021 Aug 30;18(8):e1003737. doi: 10.1371/journal.pmed.1003737 (PMC8439451; doi:10.1371/journal.pmed.1003737)
Supplement: S1 Text — (PDF) [file pmed.1003737.s001.pdf]

Public preferences for delayed or immediate antibiotic prescriptions in UK primary care: a choice experiment

Morrell et al 2021

SUPPORTING INFORMATION 1. Identification and selection of attributes

The attribute long-list was defined from a structured literature review, conducted in July 2017 to inform a series of choice studies in different contexts, one of which was this choice study. The overall aim was to generate a long-list of attributes that could potentially influence clinicians, patients, or members of the public, in giving, seeking or stopping antibiotic treatment for any condition.

Searches were restricted to studies in humans, and used the search syntax:

> (antibacterial OR anti-bacterial OR antibiotic* OR anti-infective OR antimicrobial* OR anti-microbial* OR AMR) AND (preference* OR DCE OR conjoint* OR best-worst* OR BWS OR discrete choice*)

Databases searched:

	**PubMed**	**Embase**	**Econlit**	**PsychInfo**
**Date range searched**	01/01/2005-12/02/2017	01/01/2005-12/02/2017	01/01/2005-12/02/2017	01/01/2005-13/07/2017

The literature search was undertaken in July 2017, and identified 3,066 papers. After removing duplicates, screening titles and abstracts, and then assessing full-text papers for eligibility, 89 papers were identified that met the inclusion criteria. In addition, 23 papers were identified from other sources (for example, papers that were already known to the study team). Overall, 112 papers were included.

116 potential attributes were identified in these papers. Attributes that might be important to members of the public in the context of this choice study, were identified through iterative discussion, collapsing related ideas into single attributes where necessary (for example, the attribute 'symptoms' below resulted from collapsing an attribute 'severity of symptoms' with specific symptoms such as 'colour of nasal discharge' and 'abnormal lung sounds'). 11 such attributes were then scored for importance by a convenience sample.

Table S1a: Summary of attributes as scored by convenience sample of adults

	Attributes	**Adults (N=16)**	
		**Mean score**	**Rank**
1.	Quality of communication during appointment	7.9	1
2.	Degree of benefit from antibiotics	7.8	2
3.	Whether antibiotics are indicated by a diagnostic test	7.6	3
4.	Risk of significant harm from not giving antibiotic treatment	7.3	4
5.	Symptoms	6.5	5
6.	Risk of antibiotic resistance developing	5.7	6
7.	Length of illness	5.6	7

8.	Probability that you might acquire a bacterial infection, or that infection may recur	5.2	8
9.	Number of days off work/school due to sickness	5.1	9
10.	Risk of significant harm from giving antibiotic treatment	4.9	10
11.	Cost of antibiotic prescription	2.2	11

Include; Possibly include; Exclude

Table S1b: Summary of attributes as scored by convenience sample of parents

	Attributes	Parents (N=6)	
		**Mean score**	**Rank**
1.	Risk of significant harm from not giving antibiotic treatment	8.8	1
2.	Risk of significant harm from giving antibiotic treatment	8.5	2=
3.	Quality of communication during appointment	8.5	2=
4.	Probability that your child might acquire a bacterial infection, or that infection may recur	8.4	4
5.	Whether antibiotics are indicated by a diagnostic test	8.3	5
6.	Age of child who is ill	8.3	5
7.	Symptoms	8.0	7
8.	Degree of benefit from antibiotics	7.7	8
9.	Length of illness	7.3	9
10.	Risk of antibiotic resistance developing	7.2	10
11.	Number of days off work/school due to sickness	7.0	11
12.	Cost of antibiotic prescription	0.6	12

Include; Possibly include; Exclude

We aimed to describe the choice situations using 6-8 attributes, to be acceptable to respondents without making choices excessively complex. Having decided to have the same attributes for adult and parent studies for comparability, we needed a single set of attributes that was important to both groups. We also wanted to maximise overlap between these studies and a related study among GP's, again for comparability and to identify potential differences between patients and clinicians. This meant it was possible that some attributes could be excluded despite a high importance ranking for one group, if they were less important to the others.

Diagnostic testing was important to both groups; however, there is no good test for RTIs in common use in primary care in the UK, so it was felt this attribute was unrealistic and it was not selected.

'Degree of benefit from taking antibiotics' was important to adults. However, capturing this as an attribute appeared problematic, as it is not clear how patients would know this at the time of the prescribing decision. The attribute 'Risk of harm from not giving antibiotics' captures a similar concept (the degree of benefit is in avoiding the harms from not taking antibiotics), which was more readily captured in a numerical attribute. Hence this attribute was not selected.

Similarly, 'Probability of acquiring an infection', on reflection, was problematic to execute in a DCE as a patient is unlikely to know this at the time of the prescribing decision. This attribute was not selected.

'Quality of communication', 'Risk of harm from not giving antibiotics' 'Risk of harm from giving antibiotics and 'Symptoms' were highly ranked by both groups among the remaining attributes, and 'Length of illness' was important to adults (and to GPs). 'Risk of harm from giving antibiotics' was included despite its low ranking for adults, because of its high importance to parents. Further, we described this attribute as the risk of side effects, allergy, and resistance, thus incorporating 'Risk of

antibiotic resistance developing', which was ranked sixth by adults. Comments from respondents suggested they were considering issues of resistance at an individual level – i.e. the risk of them personally acquiring a resistant infection – rather than at a population level.

'Quality of communication' was subsequently reframed in terms of the length of the appointment, as a single concept that determines the extent of explanation and discussion possible in the consultation.

'Number of days off due to sickness' was included, in a generalised form of 'disruption to usual activities', despite its low ranking in the convenience sample. This decision was made for reasons of face validity, and because we were concerned that this attribute might be important to specific population subgroups who were under-represented in our convenience sample.

The format of delivering the delayed prescription was not part of this preliminary survey. It was included for policy relevance; the formats have been tested in clinical trials and referred to in guidelines, but there are no quantitative data on patient preferences.
