# Supplementary material for: Public preferences for delayed or immediate antibiotic prescriptions in UK primary care: A choice experiment
Source: PLoS Med. 2021 Aug 30;18(8):e1003737. doi: 10.1371/journal.pmed.1003737 (PMC8439451; doi:10.1371/journal.pmed.1003737)
Supplement: S2 Text — (PDF) [file pmed.1003737.s002.pdf]

Public preferences for delayed or immediate antibiotic prescriptions in UK primary care: a choice experiment

Morrell et al 2021

SUPPORTING INFORMATION 2. Survey instrument (adult version)

A survey on attitudes towards antibiotic prescribing

Welcome to this survey.

The aim of this survey is to learn more about what people think is important when they become ill, think they may need antibiotics, and have decided to visit a doctor. We would like to invite you to participate in this study, as an adult member of the public.

We will describe some situations you might experience if you have an infection, such as a cold, sore throat, or bronchitis. We will then ask about your preferences regarding any antibiotic prescription the doctor might give you. The results will help us improve antibiotic prescribing policies for the NHS.

Health can affect, and be affected by, many different aspects of life, so we would also like to ask some questions about you, including your background and your personality. Some of these questions may not have obvious links to health, but it is important that we ask them, as your answers might help us to uncover some new and useful information that could lead to improvements in healthcare.

This survey should take around 20 minutes to complete. Some of the questions may require a little thought, but please try to answer them all if you can. There are no right or wrong answers - we are simply interested in your views.

Please note that your participation is entirely voluntary. You may withdraw at any point during the survey for any reason and without penalty, before submitting your answers, by simply closing your computer's browser window.

The survey is being carried out by researchers at the Nuffield Department of Population Health, University of Oxford. The lead researcher is Dr. Liz Morrell.

Research Now SSI is the data controller with respect to your personal data and, as such, will determine how your personal data is used. Please see their privacy notice here. Research Now SSI will share only fully anonymised data with the University of Oxford, for the purposes of research. The anonymised data will be stored on secure networks at the University of Oxford, and archived indefinitely at the end of the project.

This project has been reviewed by, and received ethics clearance through, the University of Oxford Central University Research Ethics Committee [R58252/RE003].

What if there is a problem?

Initially, please contact Research Now SSI on helpdesk_uk_ws@ssisurveys.com. If they cannot resolve your concern, they will contact the lead researcher and work with her to deal with your concern. If you remain unhappy or wish to make a formal complaint, Research Now SSI

will provide you with contact details for the Chair of the Research Ethics Committee at the University of Oxford.

Having read the information on this page, do you agree to participate in this study?

[instruction to select, then click the forward arrow]

☐ Yes, I agree to take part ☐ No, I do not agree to take part [THANK AND CLOSE]

THANK YOU FOR YOUR HELP

[screener for quotas]

1. What is your gender?

Recruit to quota

Male
Female
Other
Prefer not to say

2. How old were you on your last birthday? Please indicate your answer by entering a number in the box below:

Recruit to quota

[Numeric box] years

3. How would you describe your ethnicity or background?

Recruit to quota

White
Mixed/multiple ethnic groups
Black / African / Caribbean / Black British
Asian / Asian British
Other ethnic group
Prefer not to say

4. Which of the following best describes where you live?

Recruit to quota

South East
London
North West
East of England
West Midlands
South West
Yorkshire and the Humber
East Midlands
North East
Scotland
Wales
Northern Ireland

Thank you for that information.

The next page provides some background information about antibiotics, and describes how to complete the survey.

[new screen]

BACKGROUND

Antibiotics are medicines that are used to treat some types of infection (https://www.nhs.uk/conditions/antibiotics/). Antibiotics work by killing bacteria or preventing them from reproducing and spreading.

There are many types of infection, but in this survey we will focus on only one type: a respiratory tract infection (often abbreviated by doctors to RTI). **Respiratory tract infections are any infection affecting the nose, sinuses, throat, airways or lungs. They can be caused by viruses or bacteria, and are one of the most common reasons why people visit their GP. The 'common cold' is the most well-known example of a respiratory tract infection. Other examples include sore throat, sinusitis, bronchitis, and pneumonia.**

Some respiratory tract infections can be treated effectively with antibiotics, while others are not helped by antibiotics. In the UK, if you need antibiotics, you will require a prescription from a doctor, usually your GP. If a doctor thinks that antibiotics might be the right treatment for you, they have two options:

- They could issue a **'standard' prescription** for antibiotics for you to start taking straight away. You would usually take the prescription to a pharmacy to collect the antibiotics, and start taking them that day.

  OR

- They could issue a **'back-up' prescription** – the doctor might give you a prescription, but tell you to wait and see if your condition gets better by itself. You can collect the antibiotics and start taking them if your symptoms get worse, or if you don't feel better in a few days (the doctor will tell you how long, depending on your symptoms). If you don't feel you need the antibiotics, or your condition starts to get better by itself, then you don't need to use the prescription.

AIM OF THIS SURVEY

This survey aims to understand which type of prescription people would prefer their GP to give them, in different situations. We are interested in <u>your</u> views about what you would prefer, and what factors are important for you.

HOW TO COMPLETE THIS SURVEY

Please imagine you have a respiratory tract infection. **A respiratory tract infection is any infection affecting the nose, sinuses, throat, airways or lungs. Examples are the common cold, sore throat, sinusitis, bronchitis, and pneumonia.**

You think you might need antibiotics, and you have made an appointment to see your GP.

On the following pages you will see descriptions of various situations you might find yourself in when suffering from a respiratory tract infection. They will describe how you might be feeling, and what would be discussed during your appointment with your GP. For each situation, please choose whether you would prefer to be given a standard prescription for antibiotics that you start taking straight away, or a back-up prescription for antibiotics that you collect only if you need them.

Even if you believe you are allergic to some antibiotics, you can still complete the survey. Please assume that the doctor knows your history, and will only prescribe antibiotics that you are able to take.

Each situation is made up of <u>seven</u> features. The following pages provide more information about those features, and the choices we will ask you to make. Please read this information carefully, then answer all of the questions that follow.

THANK YOU FOR YOUR HELP

[EACH FEATURE PRESENTED ON A SEPARATE SCREEN]

Feature 1

Symptoms

This feature describes the symptoms you are experiencing when you see the GP. In each situation, the symptoms will be one of the following four options:

- sore throat and swollen glands in your neck
- sore throat, swollen glands in your neck, and fever
- chesty cough and runny nose
- chesty cough, fever and pain on breathing

Feature 2

How long you have had your current symptoms

This refers to how long you have had the symptoms, by the time you see the GP. In each situation, you will have had these symptoms for one of the following three periods:

- 3 days
- 7 days
- 10 days

Feature 3

Length of appointment with the GP

During your appointment, the doctor will listen to you describe your symptoms, ask you some questions and examine you. The doctor will then explain what he or she thinks is wrong, offer advice, and prescribe medicines if they're needed. A longer appointment provides more time for examination and discussion, allows the doctor to give you more detailed advice, and gives you more chance to ask questions.

In each situation, the length of your appointment will be one of the following three options:

- 5 minutes
- 10 minutes
- 15 minutes

Feature 4

How much longer your usual activities will be disrupted by your illness, if you don’t have antibiotics

This could include disruption of your ability to work, take care of other people, or any other of your usual activities.

Your GP would be able to give you an idea of how long people with similar symptoms usually take to feel better.

In each situation, the length of time will be one of the following four options:

- 2 days
- 5 days
- 10 days
- 14 days

Feature 5

How likely it is that you would experience harm from not having antibiotic treatment straight away

This harm might be that the symptoms start to get worse, which could happen after you had started to feel better. It could also be that the infection spreads, so you start to experience new symptoms.

Your GP would be able to give you an idea of the likelihood that you will experience harm from not having antibiotics straight away.

In each situation, the likelihood will be one of three options, illustrated in the diagrams below:

- Unlikely. For every 100 patients like you, 1 would get worse, or experience new symptoms.

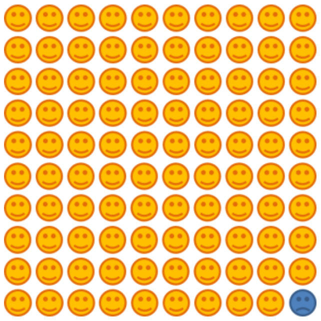

- Somewhat likely. For every 100 patients like you, 10 would get worse, or experience new symptoms.

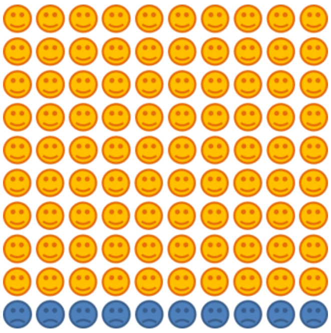

- Likely. For every 100 patients like you, 20 would get worse, or experience new symptoms.

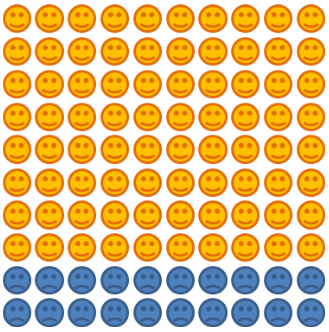

Feature 6

How likely it is that you would experience an adverse effect from <u>having</u> antibiotic treatment

An adverse effect might be an allergic reaction, or side effects such as diarrhoea or feeling sick. It could also be that next time you have an infection, it will be resistant to antibiotic treatment, so some antibiotics won't work well against it.

Your GP would be able to give you an idea of the likelihood that you will experience an adverse effect from taking antibiotics.

In each situation, the likelihood will be one of three options, illustrated in the diagrams below:

- <u>Unlikely</u>. For every 100 patients like you, 1 would experience an adverse effect.

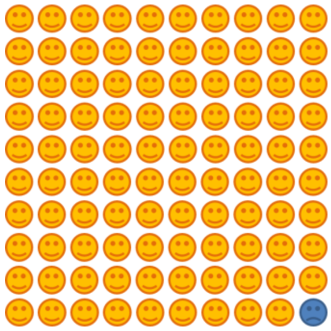

- <u>Somewhat likely</u>. For every 100 patients like you, 10 would experience an adverse effect.

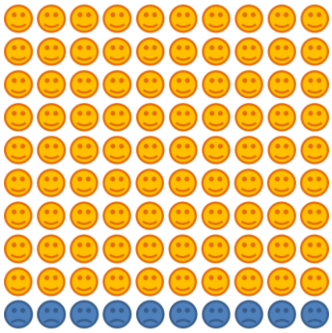

- <u>Likely</u>. For every 100 patients like you, 20 would experience an adverse effect.

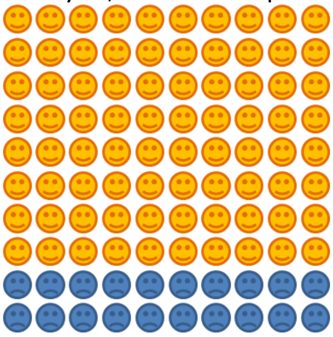

Feature 7

How your GP would issue a back-up prescription

As described before, a back-up prescription means you wait and see if you need the antibiotics. You can start taking them if your symptoms get worse, or if you don’t feel better in a few days (the doctor will tell you how long, depending on your symptoms).

GP practices have different ways of giving back-up prescriptions, if the GP decides that’s the right way to treat your infection. They differ in how much control the GP has over when you collect the medication, and how convenient it is for you to collect it.

In each situation, if your GP decided to give you a back-up prescription, it would be in one of the following ways:

- The GP would hand you a normal prescription now. They would recommend you wait, and only collect the antibiotics if your symptoms get worse, or you don’t feel better in a few days.
- The GP would hand you a prescription that shows a date in the future. You could collect the antibiotics on or after this date, if your symptoms get worse, or you don’t feel better in a few days.
- The GP would not hand you a prescription now. Your prescription would be ready for you to pick up from the practice reception - without needing to see the GP again - if your symptoms get worse, or you don’t feel better in a few days.

SUMMARY TABLE

The table below summarises the seven features and the different options that may be presented to you in each situation. Please carefully review the information in this table before proceeding.

FEATURE	POSSIBLE OPTIONS
Symptoms you are experiencing	• sore throat and swollen glands in your neck<br>• sore throat, swollen glands in your neck, and fever<br>• chesty cough and runny nose<br>• chesty cough, fever and pain on breathing
How long you have had the symptoms	• 3 days<br>• 7 days<br>• 10 days
Length of appointment with the GP	• 5 minutes<br>• 10 minutes<br>• 15 minutes
How much longer your usual activities will be disrupted by your illness	• 2 days<br>• 5 days<br>• 10 days<br>• 14 days
Likelihood of harm from <u>not having</u> antibiotics straight away	• Unlikely: for every 100 patients like you, 1 would get worse, or experience new symptoms.<br>• Somewhat likely: for every 100 patients like you, 10 would get worse, or experience new symptoms.<br>• Likely: for every 100 patients like you, 20 would get worse, or experience new symptoms.
Likelihood of an adverse effect from <u>having</u> antibiotics	• Unlikely: for every 100 patients like you, 1 would experience an adverse effect.<br>• Somewhat likely: for every 100 patients like you, 10 would experience an adverse effect.<br>• Likely: for every 100 patients like you, 20 would experience an adverse effect.
If the GP gave a back-up prescription, how would it be issued?	• GP would hand you a normal prescription now. They would recommend you wait, and only collect the antibiotics if your symptoms get worse, or you don’t feel better in a few days.<br>• GP would hand you a prescription that shows a date in the future. You could collect the antibiotics on or after this date, if your symptoms get worse, or you don’t feel better in a few days.<br>• GP would not hand you a prescription now. Your prescription would be ready for you to pick up from the practice reception - without needing to see the GP again - if your symptoms get worse, or you don’t feel better in a few days.

RANKING EXERCISE

Of the features we have just described, which ones are the most important to you?

Please rank these features below. The most important feature to you should have a ranking of 1, while the least important should have a ranking of 7.

Start by clicking on the feature that is most important to you. This will be given a ranking of 1. Then click on the second most important feature, then the third, and so on, until you have assigned a ranking to all seven features.

If you need to revise your ranking, you can click again on a feature to unselect it.

Symptoms you are experiencing
How long you have had the symptoms
Length of appointment with the GP
How much longer your usual activities will be disrupted
Likelihood of harm from not having antibiotics straight away
Likelihood of an adverse effect from having antibiotics
How your GP's practice would issue a back-up prescription

PRACTICE QUESTION

Now we would like you to complete a practice choice question, as an example of the questions you will see in the main survey.

Even if you believe you are allergic to some antibiotics, you can still complete the survey. Please assume that the doctor knows your history, and will only prescribe antibiotics that you are able to take.

Please imagine you have a respiratory tract infection. You think you might need antibiotics, and you have made an appointment to see your GP.

If a doctor thinks that antibiotics might be the right treatment for you, they have two options:

- A standard prescription, for antibiotics that you start taking straight away
- A back-up prescription, for antibiotics that you can start in a few days if you need them

Your situation is described below.

Symptoms you are experiencing	Chesty cough, fever and pain on breathing
How long you have had the symptoms	10 days
Length of appointment with the GP	5 minutes
How much longer your usual activities will be disrupted by your illness	14 days
Likelihood of harm from not having antibiotics straight away	Likely: for every 100 patients like you, 20 would experience harm
Likelihood of an adverse effect from having antibiotics	Unlikely: for every 100 patients like you, 1 would experience an adverse effect
If the GP gave a back-up prescription, how would it be issued?	GP would not hand you a prescription now. Your prescription would be ready for you to pick up from the practice reception - without needing to see the GP again - if your symptoms get worse, or you don't feel better in a few days.

In this situation, which would you prefer? Please choose one option

A standard prescription ☐

A back-up prescription ☐

MAIN QUESTIONS

Thank you for completing the ranking exercise and practice question.

Now we would like you to complete the main part of the survey.

We are going to describe 12 situations.

In all of them, we will ask you to imagine you have a respiratory tract infection, as in the practice question. However, the description of your situation will be different each time.

Even if it is very difficult to make a decision, please indicate for each situation, whether you would prefer a **standard prescription** or a **back-up prescription** for antibiotics.

QUESTION 1

Please imagine you have a respiratory tract infection. You think you might need antibiotics, and you have made an appointment to see your GP.

If a doctor thinks that antibiotics might be the right treatment for you, they have two options:

- A standard prescription, for antibiotics that you start taking straight away
- A back-up prescription, for antibiotics that you can start in a few days if you need them

Your situation is described below.

Symptoms you are experiencing	Sore throat and swollen glands in your neck
How long you have had the symptoms	10 days
Length of appointment with the GP	5 minutes
How much longer your usual activities will be disrupted by your illness	14 days
Likelihood of harm from not having antibiotics straight away	Unlikely: for every 100 patients like you, 1 would experience harm
Likelihood of an adverse effect from having antibiotics	Somewhat likely: for every 100 patients like you, 10 would experience an adverse effect
If the GP gave a back-up prescription, how would it be issued?	GP would hand you a normal prescription now. They would recommend you wait, and only collect the antibiotics if your symptoms get worse, or you don't feel better in a few days.

In this situation, which would you prefer?

A standard prescription ☐
A back-up prescription ☐

[Questions 2-12]

RANKING EXERCISE – PART TWO

Thank you for completing the main part of the survey.

Now we would like you to complete the ranking exercise again to see if your opinions have changed after answering these questions.

Please rank these features below. The most important feature to you should have a ranking of 1, while the least important should have a ranking of 7.

Start by clicking on the feature that is most important to you. This will be given a ranking of 1. Then click on the second most important feature, then the third, and so on, until you have assigned a ranking to all seven features.

If you need to revise your ranking, you can click again on a feature to unselect it.

Symptoms you are experiencing
How long you have had the symptoms
Length of appointment with the GP
How much longer your usual activities will be disrupted
Likelihood of harm from <u>not having</u> antibiotics straight away
Likelihood of an adverse effect from <u>having</u> antibiotics
How your GP's practice would issue a back-up prescription

RESPONDENT CHARACTERISTICS

Now we would like to ask some questions about you.

All of the information that you provide will help us in our analysis, and all of your details will remain confidential.

The first questions are about your experiences of antibiotics and respiratory tract infections.

1. When you were answering the choice questions, what 'adverse effect(s)' from having antibiotics were you thinking about?

   [free text]

2. To the best of your knowledge, are you allergic to any antibiotics?

   Yes
   No
   I don't know

3. To the best of your knowledge, during the last 12 months, on how many separate occasions have you yourself taken a course of antibiotics for any illness?

   [Numeric box] times

4. To the best of your knowledge, when did you yourself last have a respiratory tract infection? **(A respiratory tract infection is any infection affecting the nose, sinuses, throat, airways or lungs.)**

   I currently have a respiratory tract infection
   Within the past month
   1-6 months ago
   7-12 months ago
   More than a year ago
   I have never had a respiratory tract infection
   I don't know

5. Which of these statements best describes your awareness of back-up prescriptions, before you did this survey?

   I was fully aware
   I was aware of the term 'back-up prescription' but not sure what it meant
   I was aware that prescriptions could be given in this way, but didn't know what it was called
   I was not aware

6. Have you yourself ever been given a back-up prescription, like the ones described in this survey?

No, never
Only once
More than once
I don’t know

7. Please indicate the extent to which you agree or disagree with the following statements about antibiotics.

“Antibiotics are effective in treating infections caused by bacteria”
“Antibiotics are effective in treating infections caused by viruses”
“Bacteria can become resistant to antibiotics”
“Antibiotic resistance is an important consideration for me when deciding whether to take antibiotics”

Five options will be provided for each statement:
Agree strongly | Agree a little | Neither agree nor disagree | Disagree a little | Disagree strongly

Now we would like to ask some questions about how you see yourself.

8. There are 10 statements below. Please indicate how well each of these statements describes your personality.
“I see myself as someone who is reserved.”
“I see myself as someone who is generally trusting.”
“I see myself as someone who tends to be lazy.”
“I see myself as someone who is relaxed, handles stress well.”
“I see myself as someone who has few artistic interests.”
“I see myself as someone who is outgoing, sociable.”
“I see myself as someone who tends to find fault with others.”
“I see myself as someone who does a thorough job.”
“I see myself as someone who gets nervous easily.”
“I see myself as someone who has an active imagination.”

Five options will be provided for each statement:
Agree strongly | Agree a little | Neither agree nor disagree | Disagree a little | Disagree strongly

9. Some information is presented below using two different formats. Thinking about how you like to be given information, please choose the one you prefer.
[2 small images to be inserted, side by side, same size]

Likelihood of harm from not having antibiotics straight away	Unlikely: for every 100 patients like you, 1 would experience harm
Likelihood of an adverse effect from having antibiotics	Unlikely: for every 100 patients like you, 1 would experience an adverse effect

The GP also tells you that it is unlikely that you would experience harm from not having antibiotics straight away (for every 100 patients like you, 1 would experience harm). It is unlikely that you would experience an adverse effect from having antibiotics (for every 100 patients like you, 1 would experience an adverse effect).

10. Are you generally a person who is fully willing to take risks or do you try to avoid taking risks? Please move the slider to the appropriate point on the scale below, where 0 means “Not at all prepared to take risks” and 10 means “Fully prepared to take risks”.

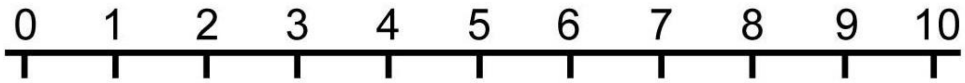

Not at all prepared to take risks

Fully prepared to take risks

We would like to end this survey by asking some questions about you.

11. Which of the following best describes your current situation?

Employed full-time
Employed part-time
Self-employed full-time
Self-employed part-time
Unemployed
Retired
Long-term sick or disabled
Looking after home or family
In full-time education
Other

12. What is the highest level of education or training you have attained?

GCSEs or ‘O’ Levels or CSEs or Scottish Standard Grade
‘A’ Levels or ‘AS’ Levels or Scottish Higher Grade
International Baccalaureate
Technical or Vocational Qualification
Transition Year Programme
Higher Education Certificate or Diploma
Undergraduate Degree
Professional Qualification (comparable to undergraduate degree)
Postgraduate Certificate or Diploma
Postgraduate Degree
Doctorate
Other
None

13. Are you currently married, in a civil partnership, or living with a partner?
    Yes
    No
    Prefer not to say

14. What is your gross annual <u>household</u> income?
    Gross household income combines your income with that of your partner or any other household members with whom you share financial responsibilities <u>before</u> any taxes or deductions.
    Up to £9,999
    £10,000 to £19,999
    £20,000 to £29,999
    £30,000 to £39,999
    £40,000 to £49,999
    £50,000 to £74,999
    £75,000 to £99,999
    £100,000 or more
    Prefer not to say

15. Do you have any dependent children who live with you? (By 'dependent' children, we mean those who are not yet financially independent).
    Yes
    No [If no, go to question 17].

16. How many dependent children do you have who live with you?
    [Numeric box]

17. Including yourself, how many adults live in your household? (This refers to all adults, including any children aged 18 or over, who live with you. Please also include any children aged 16 or 17 who live with you, if they are financially independent).
    [Numeric box]

18. How easy or difficult did you find the questions in this survey?
    Very easy
    Easy
    Quite easy
    Neither easy nor difficult
    Quite difficult
    Difficult
    Very difficult

19. Are there any further comments that you would like to make regarding this survey?
    [Free text]

You have reached the end of the survey. Thank you for taking the time to participate.
