# Supplementary material for: Public preferences for delayed or immediate antibiotic prescriptions in UK primary care: A choice experiment
Source: PLoS Med. 2021 Aug 30;18(8):e1003737. doi: 10.1371/journal.pmed.1003737 (PMC8439451; doi:10.1371/journal.pmed.1003737)
Supplement: S3 Text — (PDF) [file pmed.1003737.s003.pdf]

Public preferences for delayed or immediate antibiotic prescriptions in UK primary care: a choice experiment

Morrell et al 2021

SUPPORTING INFORMATION 3. Constraints placed on the experimental design

Constraints on which levels of specific attributes could not appear together were suggested by reviewing designs run without any constraints, and checking for implausible combinations of levels.

1. If the level for Symptoms was 'sore throat, swollen glands in your neck, and fever' then the following attribute levels were **not** permitted:
   - Duration: 10 days. It is unlikely that a patient would have been suffering a fever for such an extended period without seeking medical advice – or would have had a fever for such a length of time without the condition either resolving or progressing.
   - Appointment: 5 minutes. It is implausible that even the busiest primary care physician would spend only 5 minutes with a patient with a fever (particularly a child).
   - RiskNot: Negligible. These symptoms are suggestive of bacterial tonsillitis, so the need for antibiotics is higher than 1%.

2. If the level for Symptoms was 'chesty cough, fever and pain on breathing' then the following attribute levels were **not** permitted:
   - Duration: 10 days. It is unlikely that a patient would have been suffering a fever for such an extended period without seeking medical advice – or in fact would have had a fever for such a length of time without the condition either resolving or progressing.
   - Appointment: 5 minutes. It is implausible that even the busiest primary care physician would spend only 5 minutes with a patient with a fever (particularly a child).
   - RiskNot: Negligible. These symptoms are suggestive of bacterial pneumonia, so the need for antibiotics is higher than 1%.

3. If the level for Symptoms was 'sore throat and swollen glands in your neck' or 'chesty cough and runny nose' then the following attribute level was **not** permitted:
   - RiskNot: Likely. These symptoms are likely to be a viral sore throat or a cold. NICE Clinical Guidance CG69 says: "These conditions are largely self-limiting and complications are likely to be rare if antibiotics are withheld." Hence we avoided scenarios that presented the risk of relapse or progression with no antibiotics as 'Likely'.

Reference:

NICE Clinical Guidance CG69. Respiratory tract infections (self-limiting): prescribing antibiotics (2008). https://www.nice.org.uk/guidance/cg69, accessed 5/12/19
