# Supplementary material for: Public preferences for delayed or immediate antibiotic prescriptions in UK primary care: A choice experiment
Source: PLoS Med. 2021 Aug 30;18(8):e1003737. doi: 10.1371/journal.pmed.1003737 (PMC8439451; doi:10.1371/journal.pmed.1003737)
Supplement: S4 Text — (PDF) [file pmed.1003737.s004.pdf]

Public preferences for delayed or immediate antibiotic prescriptions in UK primary care: a choice experiment

Morrell et al 2021

SUPPORTING INFORMATION 4. Modelling the continuous variables as categorical

The models assume that the time and risk attributes can be represented as continuous variables, with a linear relationship with the outcome on the log-odds scale. In an exploratory analysis, these attributes were modelled and plotted as categorical variables. The risk and duration plots suggested a threshold effect, with the coefficient increasing with the attribute level then reaching a plateau. Alternative models with these attributes as dichotomised variables showed slightly improved model fit though the results were qualitatively very similar. However, the models exhibited collinearity due to the restructuring of the variables, and there was insufficient information to determine exactly where the cut-off should be. As a result the linear approximation was retained in the models.
