# Supplementary material for: Public preferences for delayed or immediate antibiotic prescriptions in UK primary care: A choice experiment
Source: PLoS Med. 2021 Aug 30;18(8):e1003737. doi: 10.1371/journal.pmed.1003737 (PMC8439451; doi:10.1371/journal.pmed.1003737)
Supplement: S5 Text — (PDF) [file pmed.1003737.s005.pdf]

Public preferences for delayed or immediate antibiotic prescriptions in UK primary care: a choice experiment

Morrell et al 2021

SUPPORTING INFORMATION 5. Estimation of impact on antibiotic prescribing

We take the example of sore throat, because cough is a more complex collection of acute and chronic diagnoses. Pouwels et al (1) found 59% of sore throat consultations resulted in an antibiotic prescription (so 41% got none) and the 'ideal', appropriate level of prescribing was 13%. Hence 46% of sore throat prescriptions are assumed to be inappropriate.

We assume that the 'minor' throat symptoms in the study, got no prescription. That would be consistent with the FeverPAIN tool's recommendation (score of 1). For the 'serious' sore throat symptoms, we consider a sore throat scenario that would get a FeverPAIN score of 3, for which a delayed prescribing is recommended, and which has a 34-40% probability of a streptococcal isolate.

Table S5: sore throat delayed prescribing scenario

	Base scenario + rationale	testing
SYMPTOMS	Sore throat swollen glands and fever	
DURATION	4 days	vary upwards, not 3 or below
APPOINTMENT	10 minutes	test at 5 worst case
DISRUPTION	5 days (total duration 9 days, which is reasonable)	test at 10, which would be a total duration of 14 days
RISK_NOT	20% (FeverPAIN score of 3 probability of strep is 34-40%, assume not all of these then progress and need antibiotics)	test at 5%, in line with complication rates in studies in Cochrane review
RISK_TREAT	10% (NHS website quotes 10% for common side effects and allergy)	test at 5% for conservatism
FORMAT	Prescription and recommend delaying collecting the antibiotics (most common format)	test post-dating as the most negative; collecting from reception did not have a significant effect in effects-coded model

This scenario in FeverPAIN terms is a severe sore throat, no cold, severe muscle aches, fever, duration 4-7 days, >2cm glands, moderately inflamed tonsils, and pus on tonsils. The FeverPAIN score of 3 is robust to moving from severe to moderate sore throat; dropping the pus on tonsils drops the score to 2, but the recommendation to use delayed prescribing and the probability of a streptococcal isolate remain the same. It is not robust to raising tonsil inflammation to severe, or reducing onset of illness to three days or fewer.

For 'serious sore throat', the adult study found a mean probability of 37% (95% CI: 34-39%) that respondents chose delayed prescribing. The base scenario above had a predicted probability of respondents choosing delayed prescribing of 38%, using the main-effects mixed logit model. Varying

each attribute one or two at a time as described above, gave a range of probabilities of 30-45%, with a median of 38%. Thus the 37% found in the study appears to be a reasonable estimate for a FeverPAIN score of 3.

Inappropriate immediate prescriptions where the patient could have accepted delayed: Taking this predicted probability of a patient choosing delayed prescription, we assume that for 37% of the 46% of the inappropriate prescriptions, the patient would have accepted a delayed prescription. The DESCARTE study (2, 3) found that 14% of sore throat prescriptions were delayed prescription, so we conservatively assume that 14% of these inappropriate prescriptions were already a delayed prescription. So 37 – 14 = 23% of the inappropriate prescriptions could have been additional delayed prescriptions; that is, 11% of all sore throat consultations. Of those, 69% would not have taken the antibiotics (4, 5), so an additional 7.3% of sore throat consultations would have resulted in no antibiotics being taken.

Number of sore throat consultations: Mehta et al (6) in a national survey found a community incidence of sore throats of 1.57 per person-year, and that respondents consulted their GP for 13% of sore throat illnesses, resulting in a GP consultation rate of around 0.2 per person-year. Given a UK population of 65 million, the number of sore throat consultations per year is estimated as 13 million.

Hence, the number of consultations that could have resulted in no antibiotics being taken is 7.3% x 13 million, or 949,000 prescriptions.

Sensitivity

Taking a range of probabilities included in the 95% CIs from the study (34-39%) gives a range of 9.2-11.5% of all sore throat prescriptions that could be a delayed prescription, or 825,000 to 1,032,000 prescriptions not initiated.

Taking the most extreme estimates from the model scenario (30-45%) gives a larger range of 7.4-14.3% of all sore throat prescriptions that could be delayed prescription, or 660,000 to 1,279,000 prescriptions not initiated.

If the proportion of delayed prescribing for sore throat has increased to 17% (the proportion found in our 2019 GP study (manuscript in preparation), across all RTIs), the reduction in consumed antibiotic prescriptions achieved would be estimated at 825,000 prescriptions.

The proportion of delayed prescriptions that are not actually taken in practice may be lower than the 69% observed in the trials. If we allow for double the proportion of patients to take the antibiotics (ie 38% do not take it), then the number of prescriptions avoided is 523,000.

It is possible that patients hold on to either the delayed prescription or the antibiotics, and use them on a later occasion without reconsulting. This would reduce the estimate of the number of prescriptions avoided.

References:

1. Pouwels KB, Dolk FCK, Smith DRM, Robotham JV, Smieszek T. Actual versus 'ideal' antibiotic prescribing for common conditions in English primary care. Journal of Antimicrobial Chemotherapy. 2018;73(suppl_2):19-26.
2. Little P, Stuart B, Hobbs FDR, Butler CC, Hay AD, Delaney B, et al. Antibiotic prescription strategies for acute sore throat: a prospective observational cohort study. The Lancet Infectious Diseases. 2014;14(3):213-9.
3. Little P, Hobbs FR, Moore M, Mant D, Williamson I, McNulty C, et al. PRImary care Streptococcal Management (PRISM) study: in vitro study, diagnostic cohorts and a pragmatic adaptive randomised controlled trial with nested qualitative study and cost-effectiveness study. Health Technol Assess. 2014;18(6).
4. Spurling GK, Del Mar CB, Dooley L, Foxlee R, Farley R. Delayed antibiotic prescriptions for respiratory infections. Cochrane Database Syst Rev. 2017;9:Cd004417.
5. Little P, Williamson I, Warner G, Gould C, Gantley M, Kinmonth AL. Open randomised trial of prescribing strategies in managing sore throat. BMJ. 1997;314(7082):722.
6. Mehta N, Schilder A, Fragaszy E, E. R. Evans H, Dukes O, Manikam L, et al. Antibiotic prescribing in patients with self-reported sore throat. Journal of Antimicrobial Chemotherapy. 2016;72(3):914-22.
