# Supplementary material for: Public preferences for delayed or immediate antibiotic prescriptions in UK primary care: A choice experiment
Source: PLoS Med. 2021 Aug 30;18(8):e1003737. doi: 10.1371/journal.pmed.1003737 (PMC8439451; doi:10.1371/journal.pmed.1003737)
Supplement: S6 Text — (PDF) [file pmed.1003737.s006.pdf]

Public preferences for delayed or immediate antibiotic prescriptions in UK primary care: a choice experiment

Morrell et al 2021

SUPPORTING INFORMATION 6. Attribute importance

Rankings of the seven survey attributes (Fig S6) showed symptoms and their duration to be the most important attributes to respondents, with the format of providing the delayed prescription being the least important. The main difference between adults and parents is the ranking of disruption, which is ranked third by adults (modal rank=3, mean rank = 3.91), above the risk attributes, but ranked below the risk attributes by parents (modal rank=7, mean rank = 4.90).

Fig S6: ranking of attribute importance

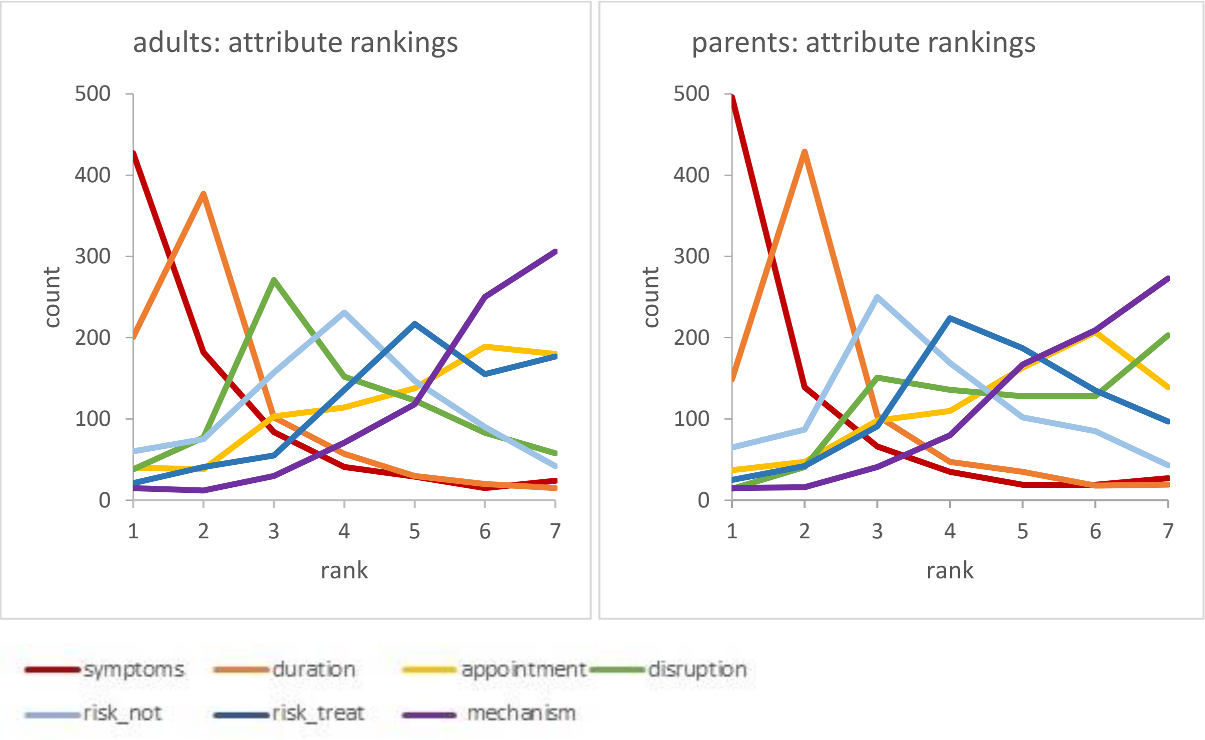

Horizontal axis: ranking from most (rank 1) to least (rank 7) importance
Vertical axis: number of respondents who gave the attribute the specified rank

risk_not = risk from not starting antibiotics immediately
risk_treat =risk of adverse effects from taking antibiotics
mechanism = format of the delayed prescription
