# Supplementary material for: Public preferences for delayed or immediate antibiotic prescriptions in UK primary care: A choice experiment
Source: PLoS Med. 2021 Aug 30;18(8):e1003737. doi: 10.1371/journal.pmed.1003737 (PMC8439451; doi:10.1371/journal.pmed.1003737)

Public preferences for delayed or immediate antibiotic prescriptions in UK primary care: a choice experiment

Morrell et al 2021

SUPPORTING INFORMATION 7. Respondent choices

15-18% of participants chose delayed prescribing for the practice question, which we had constructed using the levels most likely to indicate 'immediate' prescription.

Of the 12 choice questions, two had a roughly even split of choices for both samples (Q3 and Q8) with the most extreme preferences being ~a 70/30 split (Q4, Q6, Q11, Q12). The adult and parent samples differed most on questions 6 and 7. The highest choice of delayed prescribing was 64-67%, in Q11. Overall, adults chose delayed prescribing in 42% of choices, and parents in 44%.

Table S7: Respondent choices by question

	Question												
% chose delayed prescribing:	Practice	Q1	Q2	Q3	Q4	Q5	Q6	Q7	Q8	Q9	Q10	Q11	Q12
Adults	18	40	56	51	22	45	29	47	49	37	41	64	27
Parents	15	58	41	48	24	41	35	57	49	33	42	67	32

At the individual level, the number of times each respondent chose delayed prescribing was similar for the adult and parent samples (Fig S7). The mean number of times each sample chose delayed prescribing (5.1, 5.3 respectively) was not significantly different.

Fig S7: number of times each respondent chose delayed prescribing

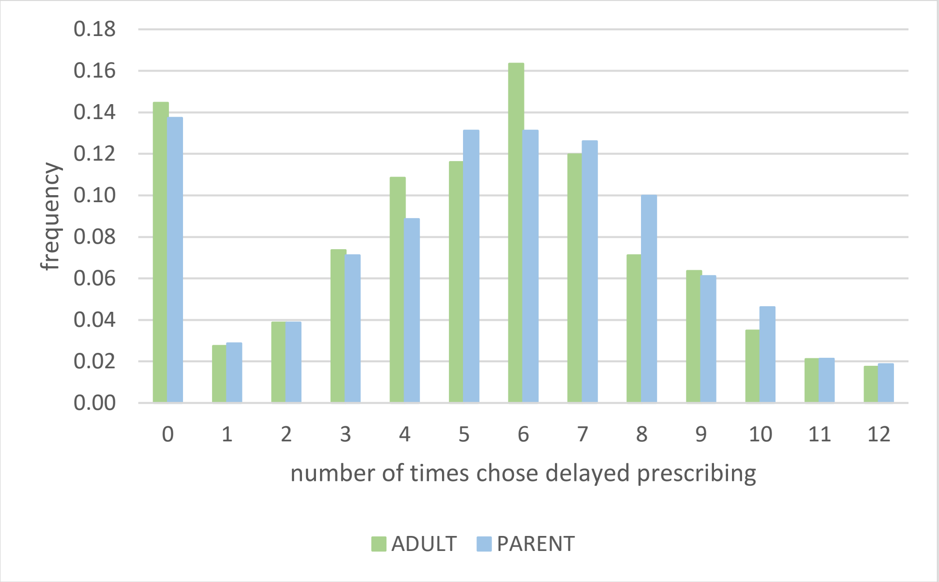

Supplement: S7 Text — (PDF) [file pmed.1003737.s007.pdf]
