# Supplementary material for: Public preferences for delayed or immediate antibiotic prescriptions in UK primary care: A choice experiment
Source: PLoS Med. 2021 Aug 30;18(8):e1003737. doi: 10.1371/journal.pmed.1003737 (PMC8439451; doi:10.1371/journal.pmed.1003737)
Supplement: S8 Text — (PDF) [file pmed.1003737.s008.pdf]

Public preferences for delayed or immediate antibiotic prescriptions in UK primary care: a choice experiment

Morrell et al 2021

SUPPORTING INFORMATION 8. Models incorporating respondent characteristics and interactions

A second pair of models incorporated respondent characteristics, and their interactions with the symptoms attribute where those interactions were significant ($p<0.05$) and policy relevant. These models showed improved fit compared to the main effects model, for both populations.

Table S8a: measures of model fit

		ADULTS (n=802)	PARENTS (n=801)
Pseudo-R^2 [a]: main effects model	Fixed effects	0.12	0.12
	Fixed and random effects	0.37	0.38
Pseudo-R^2 [a]: interactions model	Fixed effects	0.21	0.18
	Fixed and random effects	0.40	0.40
AIC, BIC [b]: main effects model		11307, 11393	11309, 11395
AIC, BIC [b]: interactions model		11067, 11325	11137, 11374

a McKelvey and Zavoina Pseudo-R^2: proportion of variability in responses that is explained by the fixed effects (attributes, respondent characteristics and interactions) in the model; and by the fixed effects plus the random intercept, which allows for individual-level variability in the tendency to choose delayed prescribing

b AIC, BIC: Akaike and Bayesian Information Criteria. A better-fitting model has lower values of these measures

Some respondent characteristics had a significant positive effect when included in the model, but the interaction was not statistically significant (for example, income and education in the adult model, number of children for parents). This indicates that respondents' overall probability of choosing delayed prescribing increased with these characteristics. These interaction terms were not retained in the final model, but the main effects were.

Where respondent characteristics showed a significant interaction with symptoms (sex, age, knowledge of antibiotics, having had no antibiotics in the past 12 months), this indicates that such groups differed in their likelihood of choosing delayed prescribing for certain symptoms. In each case, the greatest difference between subgroups was in the response to cough without fever, with some characteristics also showing differentiation between groups on sore throat without fever.

The 'knowledge' group was defined as respondents who chose 'strongly agree' for the statement that antibiotics are effective against bacteria, and 'strongly disagree' for the same statement about viruses. It is possible that some respondents who are knowledgeable about antibiotics could have chosen 'agree slightly' about bacteria, reflecting experience or understanding that particular antibiotics are not always effective against given bacteria. Using this broader definition of the 'knowledge' group ('agree slightly' as well as 'agree strongly' for effectiveness against bacteria) made little difference to the model.

Ethnicity was collapsed into three categories, to reduce the number of variables in the model and to increase the number of respondents in smaller groups. ‘mixed’ ethnicity respondents were grouped with ‘black’ ethnicity because their responses to the choice questions were most similar. Compared to an exploratory model with the original categories, this reduces the effect of ‘black’ on the probability of choosing the delayed prescription, (i.e. it is conservative in detecting an effect of this group). The models suggest cultural differences in attitudes to treating a child with a minor infection, with Asian families being less prepared to delay antibiotic treatment. It may be that there is a similar cultural difference among adults for their own treatment, which is masked by the inter-correlation between gender, age and ethnicity in this sample. These ethnicity categories are also by nature heterogeneous, and the differences between adult and parent models may be due to the specific ethnic and cultural composition of these relatively small groups in the two samples.

Table S8b: Mixed effect logit model with respondent characteristics and interactions

		ADULTS		PARENTS	
Attribute/level		Coefficient	p-value[a]	Coefficient	p-value[a]
Symptoms[b]	1	0.334	0.08	1.094	<0.001
	2	0.377	0.08	1.165	<0.001
	3	0.302	0.07	0.240	0.07
	4	Base level		Base level	
Symptom duration		-0.071	<0.001	-0.191	<0.001
Appointment length		0.006	0.32	-0.002	0.80
Disruption of usual activities		-0.072	<0.001	-0.040	<0.001
Risk of harm from not starting abx		-0.019	<0.001	-0.004	0.43
Risk of adverse effect from taking abx		0.013	<0.001	0.017	<0.001
Format of the delayed prescription[c]	1	0.176	0.003	-0.079	0.14
	2	-0.072	0.20	-0.020	0.71
	3	Base level		Base level	
Education: none		Base level		Base level	
to GCSE		0.560	0.12	-	-
post-16		0.879	0.01	-	-
degree		0.959	0.007	-	-
Income: above_£30k		0.225	0.04	-	-
Number of dependent children		-	-	0.145	0.008
Sex: male		-0.205	0.19	-0.281	0.06
SYMPTOMS x male			p(het)<0.001		p(het)=0.05
S1 x male		-0.226	0.19	-0.236	0.17
S2 x male		-0.831	<0.001	-0.485	0.007
S3 x male		-0.027	0.84	-0.189	0.17
Age group: 18 to 34		Base level		Base level	
35 to 54		-0.163	0.40	-	
55+		0.048	0.82	-	-
SYMPTOMS x age group			p(het)<0.001	-	-
S1 x age 35-54		0.276	0.19	-	-
S1 x age 55+		0.406	0.08	-	-
S2 x age 35-54		0.879	<0.001	-	-
S2 x age 55+		1.364	<0.001	-	-
S3 x age 35-54		-0.051	0.78	-	-
S3 x age 55+		-0.206	0.27	-	-
Ethnicity: white[d]		Base level		Base level	
black		-	-	0.061	0.82
asian		-	-	-0.045	0.86
SYMPTOMS x ethnicity					p(het)=0.008
S1 x black		-	-	-0.750	0.03

S1 x asian	-	-	-0.834	0.004
S2 x black	-	-	-0.549	0.08
S2 x asian	-	-	-0.856	0.003
S3 x black	-	-	-0.299	0.29
S3 x asian	-	-	-0.086	0.72
Knowledge of antibiotics[e]	-0.051	0.78	-0.204	0.28
SYMPTOMS x knowledge		p(het)<0.001		p(het)<0.001
S1 x knowledge	0.854	<0.001	1.128	<0.001
S2 x knowledge	1.331	<0.001	1.348	<0.001
S3 x knowledge	0.296	0.09	0.516	0.004
No antibiotics in past 12 months[f]	0.070	0.65	-0.094	0.52
SYMPTOMS x no_abx		p(het)<0.001		p(het)<0.001
S1 x no_abx	0.572	0.001	0.405	0.02
S2 x no_abx	0.531	0.004	0.830	<0.001
S3 x no_abx	0.224	0.13	0.265	0.05
Constant	-0.991	0.78	-0.486	0.05
var(constant)	1.747	-	1.860	-

The effect of each attribute is presented as a logistic coefficient, that is, the effect of a one unit change in the attribute, on the log odds of choosing delayed prescription.

Coefficients for categorical variables and interactions are relative to the specified base level.

x Interaction

- Characteristic is not a significant predictor of choice

a p-values given to three decimal places. p(het) is a Wald test of the interaction, which tests the null hypothesis that all the interaction coefficients are zero.

b Symptoms descriptions: 1 (S1) - sore throat and swollen glands; 2 (S2) – chesty cough and runny nose; 3 (S3) – sore throat, swollen glands and fever; 4 (S4) – chesty cough, fever and pain on breathing

c Format of delivering a delayed prescription: 1 - prescription and advice to delay collection; 2 – post-dated prescription; 3 – collect prescription from reception at a later date

d ‘white’ includes respondents who selected ‘white’ ‘other’ or ‘prefer not to say’. ‘black’ includes respondents who selected ‘black’ or ‘mixed’ ethnicity.

e Respondents who ‘strongly agreed’ that antibiotics are effective against bacteria, and ‘strongly disagreed’ that antibiotics are effective against viruses

f Respondents who reported they had not been prescribed antibiotics (adults) or had not been prescribed antibiotics for any child in their care (parents), for any illness, in the past 12 months

The above are our preferred models. For completeness we also provide a version of these models that includes all respondent characteristics and interactions from both models. The fit, interpretation and effect of the interactions is very similar.

We have not presented models that include all tested respondent characteristics. Those not included did not reach statistical significance individually as an effect in the model. When we combine more of these parameters into the model, the measures of fit (AIC, BIC) rise suggesting risk of overfitting, and we see non-convergence of the models due to correlations between the respondent characteristics.

Table S8c: measures of model fit, all respondent characteristics and interactions in both models

		ADULTS (n=802)	PARENTS (n=801)
Pseudo-R^2 [a]: main effects model	Fixed effects	0.12	0.12
	Fixed and random effects	0.37	0.38
Pseudo-R^2 [a]: interactions model	Fixed effects	0.21	0.20
	Fixed and random effects	0.40	0.40
AIC, BIC [b]: main effects model		11307, 11393	11309, 11395
AIC, BIC [b]: interactions model		11074, 11397	11135, 11458

a McKelvey and Zavoina Pseudo-R^2: proportion of variability in responses that is explained by the fixed effects (attributes, respondent characteristics and interactions) in the model; and by the fixed effects plus the random intercept, which allows for individual-level variability in the tendency to choose delayed prescribing

b AIC, BIC: Akaike and Bayesian Information Criteria. A better-fitting model has lower values of these measures

Table S8d: Mixed effect logit model, all respondent characteristics and interactions in both models

		ADULTS		PARENTS	
Attribute/level		Coefficient	p-value[a]	Coefficient	p-value[a]
Symptoms[b]	1	0.464	0.02	0.954	<0.001
	2	0.466	0.04	0.918	<0.001
	3	0.418	0.02	0.083	0.59
	4	Base level		Base level	
Symptom duration		-0.071	<0.001	-0.191	<0.001
Appointment length		0.007	0.32	-0.002	0.80
Disruption of usual activities		-0.072	<0.001	-0.040	<0.001
Risk of harm from not starting abx		-0.019	<0.001	-0.005	0.42
Risk of adverse effect from taking abx		0.013	<0.001	0.017	<0.001
Format of the delayed prescription[c]	1	0.177	0.003	-0.080	0.14
	2	-0.072	0.20	-0.021	0.71
	3	Base level		Base level	
Education: none		Base level		Base level	
to GCSE		0.525	0.15	1.013	0.36
post-16		0.862	0.02	1.324	0.23
degree		0.954	0.008	1.328	0.23
Income: above_£30k		0.213	0.06	0.137	0.27
Number of dependent children		0.054	0.33	0.120	0.03
Sex: male		-0.209		-0.308	0.04
SYMPTOMS x male			p(het)<0.001		p(het)=0.02
S1 x male		-0.213	0.21	-0.273	0.12
S2 x male		-0.827	<0.001	-0.558	0.002
S3 x male		-0.013	0.93	-0.228	0.10
Age group: 18 to 34		Base level		Base level	
35 to 54		-0.147	0.45	-0.060	0.70
55+		0.118	0.59	-0.259	0.51
SYMPTOMS x age group			p(het)<0.001		p(het)=0.27
S1 x age 35-54		0.194	0.36	0.277	0.14
S1 x age 55+		0.294	0.21	0.143	0.78
S2 x age 35-54		0.828	<0.001	0.496	0.01
S2 x age 55+		1.291	<0.001	0.295	0.54
S3 x age 35-54		-0.130	0.49	0.306	0.05
S3 x age 55+		-0.310	0.12	0.276	0.41
Ethnicity: white[d]		Base level		Base level	
black		0.450	0.20	0.090	0.75
asian		-0.188	0.55	-0.086	0.74
SYMPTOMS x ethnicity			p(het)=0.28		p(het)=0.01
S1 x black		-0.753	0.08	-0.763	0.03

S1 x asian	-0.112	0.73	-0.784	0.008
S2 x black	-0.327	0.50	-0.572	0.06
S2 x asian	-0.223	0.57	-0.774	0.009
S3 x black	-0.808	0.04	-0.313	0.28
S3 x asian	0.015	0.96	-0.027	0.91
Knowledge of antibiotics[e]	-0.058	0.74	-0.229	0.22
SYMPTOMS x knowledge		p(het)<0.001		p(het)<0.001
S1 x knowledge	0.845	<0.001	1.108	<0.001
S2 x knowledge	1.323	<0.001	1.311	<0.001
S3 x knowledge	0.293	0.09	0.490	0.006
No antibiotics in past 12 months[f]	0.093	0.54	-0.087	0.56
SYMPTOMS x no_abx		p(het)=0.01		p(het)<0.001
S1 x no_abx	0.544	0.001	0.359	0.04
S2 x no_abx	0.512	0.006	0.750	<0.001
S3 x no_abx	0.199	0.18	0.211	0.12
Constant	-1.054	0.01	-1.702	0.13
var(constant)	1.746	-	1.81863	-

The effect of each attribute is presented as a logistic coefficient, that is, the effect of a one unit change in the attribute, on the log odds of choosing delayed prescription.

Coefficients for categorical variables and interactions are relative to the specified base level.

x Interaction

a p-values given to three decimal places. p(het) is a Wald test of the interaction, which tests the null hypothesis that all the interaction coefficients are zero.

b Symptoms descriptions: 1 (S1) - sore throat and swollen glands; 2 (S2) – chesty cough and runny nose; 3 (S3) – sore throat, swollen glands and fever; 4 (S4) – chesty cough, fever and pain on breathing

c Format of delivering a delayed prescription: 1 - prescription and advice to delay collection; 2 – post-dated prescription; 3 – collect prescription from reception at a later date

d ‘white’ includes respondents who selected ‘white’ ‘other’ or ‘prefer not to say’. ‘black’ includes respondents who selected ‘black’ or ‘mixed’ ethnicity.

e Respondents who ‘strongly agreed’ that antibiotics are effective against bacteria, and ‘strongly disagreed’ that antibiotics are effective against viruses

f Respondents who reported they had not been prescribed antibiotics (adults) or had not been prescribed antibiotics for any child in their care (parents), for any illness, in the past 12 months
