# Supplementary material for: Public preferences for delayed or immediate antibiotic prescriptions in UK primary care: A choice experiment
Source: PLoS Med. 2021 Aug 30;18(8):e1003737. doi: 10.1371/journal.pmed.1003737 (PMC8439451; doi:10.1371/journal.pmed.1003737)
Supplement: S1 Checklist — (PDF) [file pmed.1003737.s009.pdf]

Conjoint Analysis Applications in Health—a Checklist: A Report of the ISPOR Good Research Practices for Conjoint Analysis Task Force

1. Was a well-defined research question stated and is conjoint analysis an appropriate method for answering it?	
1.1 Were a well-defined research question and a testable hypothesis articulated?	Introduction para 3; Methods para 1
1.2 Was the study perspective described, and was the study placed in a particular decision-making or policy context?	Introduction para 1, 2, 4
1.3 What is the rationale for using conjoint analysis to answer the research question?	Introduction para 5
2. Was the choice of attributes and levels supported by evidence?	
2.1 Was attribute identification supported by evidence (literature reviews, focus groups, or other scientific methods)?	Methods/Defining attributes and levels, para 1; Supplementary Material SM1
2.2 Was attribute selection justified and consistent with theory?	Methods/Defining attributes and levels, para 2; Supplementary Material SM1
2.3 Was level selection for each attribute justified by the evidence and consistent with the study perspective and hypothesis?	Methods Table 1
3. Was the construction of tasks appropriate?	
3.1 Was the number of attributes in each conjoint task justified (that is, full or partial profile)?	Methods/Defining attributes and levels, para 2
3.2 Was the number of profiles in each conjoint task justified?	Methods/Choice questions para 2
3.3 Was (should) an opt-out or a status-quo alternative (be) included?	Methods/Choice questions para 1
4. Was the choice of experimental design justified and evaluated?	
4.1 Was the choice of experimental design justified? Were alternative experimental designs considered?	Methods/Survey and experimental design para 3
4.2 Were the properties of the experimental design evaluated?	Methods/Survey and experimental design para 3
4.3 Was the number of conjoint tasks included in the data-collection instrument appropriate?	Methods/Survey and experimental design para 1 (Section 2)
5. Were preferences elicited appropriately, given the research question?	
5.1 Was there sufficient motivation and explanation of conjoint tasks?	Methods/Choice questions para 1; /Survey and experimental design para 1 (Section 1)
5.2 Was an appropriate elicitation format (that is, rating, ranking, or choice) used? Did (should) the elicitation format allow for indifference?	Methods/Choice questions para 2
5.3 In addition to preference elicitation, did the conjoint tasks include other qualifying questions (for example, strength of preference, confidence in response, and other methods)?	Methods/Survey and experimental design para 1 (Section 2)

(ISPOR checklist continued)

6. Was the data collection instrument designed appropriately?	
6.1 Was appropriate respondent information collected (such as sociodemographic, attitudinal, health history or status, and treatment experience)?	Methods/Survey and experimental design para 1 (Section 3)
6.2 Were the attributes and levels defined, and was any contextual information provided?	Methods/Survey and experimental design para 1 (Section 2);Supplementary Material SM2
6.3 Was the level of burden of the data-collection instrument appropriate? Were respondents encouraged and motivated?	Methods/Survey and experimental design para 2; /Data collection
7. Was the data-collection plan appropriate?	
7.1 Was the sampling strategy justified (for example, sample size, stratification, and recruitment)?	Methods/Survey and experimental design para 4; /Data collection
7.2 Was the mode of administration justified and appropriate (for example, face-to-face, pen-and-paper, web-based)?	Methods/Data collection
7.3 Were ethical considerations addressed (for example, recruitment, information and/or consent, compensation)?	Methods para 2
8. Were statistical analyses and model estimations appropriate?	
8.1 Were respondent characteristics examined and tested?	Results/Respondent characteristics para 1-3
8.2 Was the quality of the responses examined (for example, rationality, validity, reliability)?	Methods/Analysis para 2; Results/Choice responses; /Choice modelling para 5
8.3 Was model estimation conducted appropriately? Were issues of clustering and subgroups handled appropriately?	Methods/Analysis para 1, 3; Table 1; Supplementary Material SM6; Results/respondent characteristics and interactions; Discussion para 5-10
9. Were the results and conclusions valid?	
9.1 Did study results reflect testable hypotheses and account for statistical uncertainty?	Results/Choice modelling para 1-5
9.2 Were study conclusions supported by the evidence and compared with existing findings in the literature?	Discussion para 1-10
9.3 Were study limitations and generalizability adequately discussed?	Discussion para 11-15
10. Was the study presentation clear, concise, and complete?	
10.1 Was study importance and research context adequately motivated?	Introduction para 1; Discussion para 4, 9-10
10.2 Were the study data-collection instrument and methods described?	Methods/Survey and experimental design; Supplementary Material SM2
10.3 Were the study implications clearly stated and understandable to a wide audience?	Author Summary section

Reference:

Bridges JFP, Hauber AB, Marshall D, Lloyd A, Prosser LA, Regier DA, et al. Conjoint Analysis Applications in Health—a Checklist: A Report of the ISPOR Good Research Practices for Conjoint Analysis Task Force. Value in Health. 2011;14(4):403-13
