# Supplementary material for: Public preferences for delayed or immediate antibiotic prescriptions in UK primary care: A choice experiment
Source: PLoS Med. 2021 Aug 30;18(8):e1003737. doi: 10.1371/journal.pmed.1003737 (PMC8439451; doi:10.1371/journal.pmed.1003737)
Supplement: Attachment — Submitted filename: morrell.pdf [file pmed.1003737.s010.pdf]

This paper describes a choice experiment into UK preferences for modes of antibiotic prescription.

I was asked for a statistical report and I interpret that to include all aspects of the design and conduct of the study.

Points of detail

Page 9 I can see why the authors did not include the option of no prescription but this does seem to rather limit the generalisability of their findings. Does this need more discussion later?

Page 10 I think I might emphasise here the fact that the hypothesised odds ratios apply per unit of the continuous variables. I know that if the reader refers back to footnote b of Table 1 they will realise that fact but it is easy to miss and 1.01 is quite a small odds ratio to be a minimal important difference for a categorical variable.

Page 11 I might add the phrase ‘with a random intercept for respondent’ somewhere in the first paragraph.

Page 11 The phrase ‘for each level of the categorical variables’ implies there was more than one but in Table 1 I only count one. This is confirmed in Table 3.

Page 11 I think readers in health journals would be more used to the phrase random slope models or random coefficient models than random effect models. Perhaps this depends on what your favourite software calls them.

Page 12 I wonder whether for an international readership the UK median household income would be helpful. This is one of the few occasions where I might also give the modal category since it is different (rather to my surprise).

Page 16 It is curious that at least some parents (33) stated that they had received a delayed prescription but claimed not to be aware of them.

Page 20 I am not sure this graphic adds very much over and above the actual numbers in the text. How about showing the actual distribution of the predicted probabilities?

Page 21 I assume respondent age refers to parent’s age not child’s age?

Page 25 Making claims of uniqueness is a hostage to fortune.

Supplementary material Figure S1 Should this be in the main body of the article? If it was worth getting the respondents to do the ranking presumably it is worth a bit more prominence.

Supplementary material SM6 Yes, I agree

Supplementary material Table S4 It would be better to give all the coefficients, even those which correspond to overall effects which fail to reach statistical significance for the benefit of later workers who repeat this and want to compare.

I felt that back–up was a better description than delayed and I notice it is used in the material for respondents. In the scenario where I am given a script but advised not to get it filled until I need it it is not the script which has been delayed.

Points of more substance

Models and interactions

On page 28 the authors state that the attributes were collinear. This makes it seem rather accidental but if you select 12 points in $\mathbb{R}^7$ you have whole areas of the space unrepresented and so it is not surprising that you cannot examine interactions. I think expressing it the way the authors have rather understates the seriousness of what Bellman called the curse of dimensionality (`https://en.wikipedia.org/wiki/Curse_of_dimensionality`). Even if we restrict ourselves to the levels chosen there are $4 \times 3 \times 3 \times 4 \times 3 \times 3 \times 3 = 3888$ points to choose from. I am not sure there is much practically that can be done here and I accept that some regions of the space can in this case be ruled out. The authors suggestion of using some form of incomplete blocks needs stressing here for the benefit of future workers.

Generalisability

The vignettes suppose that the patient is presenting with the expectation of receiving an antibiotic and is presenting to a doctor who would prescribe one. Would we expect the results to apply to patients and doctors who would not have those expectations?

Summary

Mostly points for clarification and emphasis.

Michael Dewey
