# Supplementary material for: Public preferences for delayed or immediate antibiotic prescriptions in UK primary care: A choice experiment
Source: PLoS Med. 2021 Aug 30;18(8):e1003737. doi: 10.1371/journal.pmed.1003737 (PMC8439451; doi:10.1371/journal.pmed.1003737)
Supplement: Attachment — Submitted filename: response to reviewers v2.docx [file pmed.1003737.s011.docx]

**RESPONSES TO REVIEWER FEEDBACK**

**Reviewer #1 - Statistical review**

Points of detail

Page 9 I can see why the authors did not include the option of no prescription but this does seem to rather limit the generalisability of their findings. Does this need more discussion later?

Thank you for raising this, which was an important consideration in the design of the study. As suggested we have added some discussion of this point to the Discussion (paragraph 14), considering how adding a ‘no prescription’ option could have affected our results; we conclude that our design may provide a conservative estimate of the acceptability of alternatives to an immediate prescription.

Page 10 I think I might emphasise here the fact that the hypothesised odds ratios apply per unit of the continuous variables. I know that if the reader refers back to footnote b of Table 1 they will realise that fact but it is easy to miss and 1.01 is quite a small odds ratio to be a minimal important difference for a categorical variable.

We have added this clarification in the paragraph on sample size (Methods, under ‘Survey and experimental design’, paragraph 4).

Page 11 I might add the phrase `with a random intercept for respondent' somewhere in the first paragraph.

We have added this phrase; we had tried originally to avoid too much statistical terminology, but accept your point below about readers of health journals being familiar with this vocabulary (Methods, under ‘Analysis’, paragraph 1).

Page 11 The phrase `for each level of the categorical variables' implies there was more than one but in Table 1 I only count one. This is confirmed in Table 3.

There are two categorical variables – the patient’s symptoms, and the format of the delayed prescription. We have clarified this sentence (Methods, under ‘Analysis’, paragraph 1), and made the tables clearer.

Page 11 I think readers in health journals would be more used to the phrase random slope models or random coefficient models than random effect models. Perhaps this depends on what your favourite software calls them.

We have made this change (Methods, under ‘Analysis’, paragraph 3)

Page 12 I wonder whether for an international readership the UK median household income would be helpful. This is one of the few occasions where I might also give the modal category since it is different (rather to my surprise).

We have added the comparison to the UK median income, and a comment on the difference in modes (Results, under ‘Respondent characteristics’, paragraph 2).

Page 16 It is curious that at least some parents (33) stated that they had received a delayed prescription but claimed not to be aware of them.

We agree with the reviewer that this is interesting, although we note that around half of these respondents were classified as ‘partially aware’ in the awareness question (aware of the term, or that prescriptions could be delivered in this way). Nevertheless, there remain 17 who have had a delayed prescription but claim to have been unaware of them before completing the survey. Our explanation is that having completed the survey, they may have realised that a prescription they had in the past did indeed follow this description, although they had thought little of it before being asked this question. An add-on ‘thinkaloud’ study would help to understand this better. Given the length of the manuscript, we have not added further to the main text on this, but could do so if the Reviewer/Editors felt it would be helpful.

Page 20 I am not sure this graphic adds very much over and above the actual numbers in the text. How about showing the actual distribution of the predicted probabilities?

We have re-considered this graph, and agree it adds little to the text. Further, Reviewer 3 found it confusing. We have deleted the graph.

Page 21 I assume respondent age refers to parent's age not child's age?

Yes, you are correct. We have clarified the text (Results, under ‘Respondent characteristics and interactions’, paragraph 3)

Page 25 Making claims of uniqueness is a hostage to fortune.

We have revised this sentence to state that it is the first UK study to quantify the trade-offs, to the best of our knowledge (Discussion, paragraph 4)

Supplementary material Figure S1 Should this be in the main body of the article? If it was worth getting the respondents to do the ranking presumably it is worth a bit more prominence.

We have added a paragraph summarising the attribute rankings (Results, under ‘Attribute importance’).

Supplementary material Table S4 It would be better to give all the coefficients, even those which correspond to overall effects which fail to reach statistical significance for the benefit of later workers who repeat this and want to compare.

Thank you for this guidance. All the main effects were included in all models, but our preferred models (Table S4) omit respondent effects or interactions that did not reach significance at a p<0.05 threshold. For completeness, we have added an alternative model (Table S4a) which includes all respondent characteristics and interactions that were significant for either sample, in a common model. The coefficients and measures of fit are very similar to our more parsimonious version.

When we add in more of the characteristics that did not reach significance in either model when tested individually, we start to run into difficulties with non-convergence due to correlations between respondent characteristics, and the measures of fit (AIC, BIC) rise suggesting a risk of over-fitting. We have added this clarification to the text in the Supplementary Material (re-named Supporting Information) Section 8.

I felt that back-up was a better description than delayed and I notice it is used in the material for respondents. In the scenario where I am given a script but advised not to get it filled until I need it it is not the script which has been delayed.

We discussed this extensively at the design stage, as there is no one single definition for the terms 'delayed' and 'back-up'. Indeed, NICE uses both terms interchangeably (for example, see <https://www.nice.org.uk/guidance/qs121/chapter/Quality-statement-2-Back-up-delayed-prescribing>). Past studies have also used other terms such as ‘wait-and-see' and ‘just-in-case'. Importantly, the term 'back-up' is also used for patients with chronic conditions who may be given a 'back-up' prescription or 'rescue pack' for antibiotics for (for example) a potential future acute exacerbation of COPD. These situations are outside the scope of our study as we focused on the use of delayed/back-up prescription for a current acute infection.

We agree that 'back-up' may be a more intuitive description in general hence we chose to use this term in our survey. However, given the prevalence of existing literature in this area using the term ‘delayed’, and to avoid confusion with the other usage, we chose to use the term 'delayed' in this paper. This is consistent with NICE’s terminology, and we felt that the term would be recognisable to journal readers.

Points of more substance

Models and interactions: On page 28 the authors state that the attributes were collinear. This makes it seem rather accidental but if you select 12 points in R^7^ you have whole areas of the space unrepresented and so it is not surprising that you cannot examine interactions. I think expressing it the way the authors have rather under-states the seriousness of what Bellman called the curse of dimensionality (https://en.wikipedia.org/wiki/Curse_of_dimensionality). Even if we restrict ourselves to the levels chosen there are 4x3x3x4x3x3x3 = 3888 points to choose from. I am not sure there is much practically that can be done here and I accept that some regions of the space can in this case be ruled out. The authors suggestion of using some form of incomplete blocks needs stressing here for the benefit of future workers.

Thank you for raising this important point, which in retrospect we did not express clearly in the original submission. We have now explicitly noted that the difficulties with collinearity were with the introduction of interaction terms, rather than with the main effects estimation (Discussion, paragraph 13). We have also commented on this point in the limitations section of the Discussion, on the issue of sampling from 3888 points in 7-dimensional space. We did use experimental design software to optimise the design, and agree that some can be ruled out. An incomplete block design in future work would help to address both of these points, as noted in the Discussion (amended for clarity in the revision).

Generalisability: The vignettes suppose that the patient is presenting with the expectation of receiving an antibiotic and is presenting to a doctor who would prescribe one. Would we expect the results to apply to patients and doctors who would not have those expectations?

Thank you for this point on the generalisability of our findings. We set the expectation that the patient believes they need antibiotics in order to firstly justify the decision to be consulting with a doctor (expecting that individuals may have different thresholds for consultation), and second to be internally consistent with the choice not to have a ‘no prescription’ option. In a sample where this had not been set, we suggest that their choices would reflect a lower demand for antibiotics. In that case, our results are likely to be conservative in estimating the acceptance of antibiotic avoidance.

Reviewer 2 raised a similar point regarding the role of the doctor. Our study makes no assumptions about the doctor’s behaviour, but asks the respondent what they would prefer given the information in the scenario. Our estimate of the consumption impact assumes that the doctor uses delayed prescribing where current levels of immediate prescription are inappropriate, and that the patient accepts the recommendation. Further work is needed to understand the beliefs and incentives affecting the doctor’s behaviour. We have added this comment to the Discussion (paragraph 15).

**Reviewer #2:**

This is a generally well written DCE on an important topic. The below comments are meant to be constructive.

This is possibly a reflection of my understanding but I don’t entirely understand the design. Normally in a DCE different profiles are assembled for different options eg A v B, but here it isn’t the case. The A v B (or labels) are still there but the attributes are the same across the profiles. For example, someone is going to be ill for 14 days irrespective of choice (In the example)? How then is the independent importance of this attribute determined, given it hasn't been rotated? Additionally, in this sense length of symptom for example is not a design attribute (of the AntiB itself), its background factor. I would suggest that as a minimum the design / analysis approach should be better explained.

We accept that our description of the design was not sufficiently clear. We have added an additional paragraph to the Methods section (under ‘Choice questions’) to improve our explanation of the design, analysis and expected outputs.

As you point out, in contrast to most common DCE designs, we do not present two profiles for respondents to choose between, but a single profile and two prescription options. In our design, the analysis estimates the effect of each attribute on the probability of choosing the delayed prescription option – that is, a non-conditional binary outcome, hence we use a logistic regression.

The attributes are, as the reviewer notes, intended to be independent of the response chosen by the respondent. If they might be expected to be affected by treatment choice (for example, expected duration of disruption of activities due to illness), they are explained in the introduction to the survey, as being the expectation if the person did not have immediate antibiotics. The respondent’s beliefs about antibiotics – which is what we are aiming to elicit - will then determine whether they decide that immediate antibiotics are needed, or a delayed prescription would be acceptable.

Given this is effectively a labelled design, is there a constant in the model to reflect the independent preference for one of the two option (ie the alternative specific constant)?

There is a constant, which we have added into Table 3. It is not the alternative-specific constant (ASC) estimated in the conditional logistic regression usually used to analyse DCEs, but a logistic regression constant, reflecting the odds of the modelled outcome (in this case, the delayed prescription) when all parameters are simultaneously zero. It is therefore not as readily interpretable as the ASC. We have included an explanation of the two types of constant in the Methods, under ‘Choice questions’, and added an explanatory footnote to Table 3.

I have to say I struggled with the meaning and logic of some of the design attributes. For example, the attribute about length of interruption is strange, as this will not be known with certainty? Therefore how can it be specified in advance. Also wouldn’t this be correlated with symptoms?

We have improved the clarity of our description of the attributes in Table 1. The attributes were explained to respondents in the introduction to the survey, which is provided in the Supporting Information. We have added more of this material to Table 1. For example, the duration of disruption attribute was explained as how long people with similar symptoms would normally take to feel better, and that the doctor would be able to give the patient an idea of how long that would usually be; resources such as the TARGET patient leaflet include expected durations. Similarly the doctor would be able to give them an idea of the likelihood of negative outcomes due to either delaying antibiotics, or from taking them.

It is possible that there would be correlation between the attributes, and therefore some combinations will be implausible if the attribute levels were purely randomly assorted. We excluded some of the most obviously implausible combinations (for example, a minor sore throat was not allowed to occur in a scenario with a 20% risk of harm from delaying antibiotics). However, if we had excluded large numbers of combinations, the levels would have occurred repeatedly in the same combinations, reducing our ability to estimate their individual effects. Hence as with any experiment of this type, there may be combinations that are perhaps unlikely, but still imaginable for the respondent. We have noted in the limitations that the hypothetical scenarios may not exactly reflect respondents’ experience of real-life respiratory tract infections (RTIs) (Discussion, paragraph 11).

Risk of harm is very ambiguous and a resistance as terms / concepts - what could they imply in terms of impact on health? No or little information is given about this.

The percentages for risks of harm due to delay were based on placebo data from antibiotic studies in RTIs. Although specific medical complications occurred at low frequency, ‘harms’ as described for our respondents included persistence, recurrence, and new symptoms (now added to the description in Table 1). Incidence of these outcomes in the studies was in some cases in the high teens.

Also for a DCE to work, participants must trade off attributes. So there must be an upside and downside to a choice. Here I struggled with the design in so much that I am not clear what is meant by the 'risk of harm' or adverse effect of taking an AB, or how often they occur. 20% seems very high - is this plausible?

As you describe, there must be a trade-off. In this design it is at the level of the binary decision: for example, these symptoms have been present for a long time (suggesting I need immediate antibiotics) but the downside is the high risk of adverse events (suggesting I’d rather delay). The respondent’s choice reflects the relative importance of the attributes.

It strikes me as important that no prescription was omitted as an option. If it had been included, could it have impacted the results. That is, if the alternative was not receiving anything, then presumably a delayed prescription would be more appealing?

Thank you for raising this point (as did Reviewer 1), which was an important part of our discussions at the design stage of this study. Our research question was to determine the factors favouring delayed prescription rather than immediate. Adding a ‘no prescription’ option would have been a valid question in itself, but we had chosen to focus on delayed prescription and we were concerned that we would lose information on that specific question, by offering a ‘no prescription’ option. We then framed the choice questions to be internally consistent with the choices offered, such that respondents were expecting that they would be given a prescription (‘you think you might need antibiotics’). We have expanded our explanation of this rationale in the Methods (under ‘Choice questions’)

It is possible that, as you describe, including a ‘no prescription’ option could have increased the proportion choosing delayed prescription rather than immediate. Were that the case, then our experiment as presented would be a conservative estimate of the acceptability of delayed prescribing. We have added this discussion to the limitations section in the Discussion (paragraph 14).

I would suggest an OR of 0.997 is equivalent to 1 for all practical purposes, hence I wouldn't describe the attribute as having a modest impact.

We have corrected the sentence to describe this as providing no evidence of an effect (Results, under ‘Choice modelling’, paragraph 2).

I am very surprised that the format of the delayed prescription didn't have any impact. I would have thought going back to the practice would have been seen as annoying and unnecessary (on the premise that people usually highly value their time)?

We were also surprised that there was no evidence for an effect of the format among parents. The larger effects of the symptoms and duration attributes suggest that parents were focused on these, and paid less attention to the format of the delayed prescription. This may not reflect the reaction they would have in reality to being asked to return to the practice. We have added this comment to the Discussion (paragraph 7).

The paper assumes people who receive a delayed prescription do not take them if not required. But surely an issue with this approach is that they might be kept and used a later date (without first seeking medical advice)?

The question of ‘leftover’ antibiotics is an interesting one. Studies across Europe (Grigoryan et al, 2007) and specifically in the UK (McNulty et al, 2019) found low reported levels of use of leftover antibiotics (0.6%, 1.3% of the respective samples); however, neither of these studies explored the role of delayed prescription in this behaviour. The actual prescription will typically only be valid for a limited time period, so cannot be held indefinitely for future collection. We believe it is unlikely that possible future use would affect choices in the study; there may be an effect on consumption, and we have noted this in our estimate of consumption reduction (Supporting Information, Section 5).

Even a person was willing to delay, it might not be medically appropriate, therefore where does this leave the results?

Thank you for raising this question, reflecting the role of the doctor as the person responsible for the prescribing decision. Reviewer 1 raises a related point. Our study makes no assumption about the doctor’s behaviour, but asks the respondent what they would prefer given the information in the profile. We were interested in acceptability to patients, as patient response can potentially affect prescribing decisions, particularly if GPs are reluctant to use delayed prescribing because of concerns about patient acceptance.

Our estimate of the antibiotic consumption impact assumes that the doctor uses delayed prescribing where current immediate prescription is inappropriate, and that the patient accepts the recommendation. If the doctor were to choose not to prescribe at all, our estimate of consumption impact would be conservative. Further work is needed to understand the beliefs and incentives affecting the doctor’s behaviour. We have added this comment to the Discussion (paragraph 15).

How was the sample size estimate calculated?

The sample size estimation used the method of de Bekker-Grob *et al* (2015), which uses the estimated variance from the experimental design, the expected effect size, and the required power and significance level. This is a widely used method to create sample sizes for choice surveys. We have expanded the description provided in the Methods (under Survey and experimental design, final paragraph) in addition to the reference.

Are respondents paid to be complete the survey, if so what biases could this introduce?

Respondents have signed up to be part of an online panel, and are rewarded for survey participation with loyalty points. In this case the points have a nominal value of around £1, in line with similar length surveys with this provider. We have no basis to judge the scale or direction of any bias due to this small payment. For transparency we have added the details to the Methods, under ‘Data collection’.

**Reviewer #3:**

Comments to the authors

Overall this is an interesting study on a relevant topic, i.e. the acceptability of delayed prescription and the underlying drivers for acceptance by the general public.

This study complements nicely earlier work done on the potential role of delayed prescriptions in the decrease of outpatient antibiotic use for common pathologies. Its results may serve also as valuable background information to feed into educational programmes and/or more targeted use of delayed prescriptions.

In general the study appears to have been designed, planned and carried out meticulously.

Methods

This section is very detailed and convincing that the authors designed their study very meticulously. A few remarks:

-survey instrument: among the survey developers, I did not notice a pharmacist nor a pediatrician. I think this is a pity as both may play an important role in the creation or carrying out of delayed prescriptions

Apologies, we did have a pharmacist in the review group, who provided insights about patient reactions and ways of explaining delayed prescription. We have now mentioned this pharmacist in the text (Methods, under ‘Survey and experimental design’, paragraph 1). We did not include a paediatrician, relying on the GPs for insight into consultations with parents with a sick child since GPs see both adult and paediatric patients in the UK. We have clarified this in the Methods since we appreciate this is not necessarily the case in other countries.

-population surveyed: in both groups there is a clear predominance (87/83%) of white people from British origin. Also, 81% of the respondents lived with their partner, and on average the population was more educated than average. This may not reflect the reality in many poor inner city neighbourhoods, with higher percentages of ethnics groups in challenging socio-economical situations, single parents etc… Migrants may have yet very different views and understanding on the use of antibiotics. This discrepancy may cause a considerable bias when it comes to drawing conclusions and designing a strategy for further interventions. Perhaps this study should be repeated (maybe with other methodologies) targeting these risk groups.

We fully agree with this point about generalisability, and have added a paragraph to the limitations section in the Discussion (paragraph 12).

Results

Table 2b: 15% of the adults mention a problem of 'allergy'. I am surprised the authors did not mention this in their discussion, as it is well known that presumed allergy is very often not real, but is an important reason for inappropriate use of second line antibiotics with even more side effects and selection of resistance (e.g. fluoroquinolones).

We have added a comment in our discussion of the respondent characteristics (Results, under ‘Respondent characteristics’, paragraph 3). Our expectation is that the person’s belief that they are allergic will affect their choices, even if the allergy is not in fact real; we have therefore taken these self-reports at face value for the analysis.

Ln 272: the difference between 'delayed' and a 'post-dated prescription' is not fully clear to me as a reader. Please define clearly and add to the method's section

We have added a fuller explanation to Table 1 in the Methods section.

Ln 281: the findings from Figure 1 appear to be discordant with the Odd's ratio's mentioned in Table 3. Please explain how you obtained these results and how to explain these differences.

Table 1 shows the odds ratios estimated in the regression model. Figure 1 uses the model to predict the probability that a respondent shown a scenario with a given description of the symptoms, would choose the delayed prescription. As Reviewer 1 points out, these probabilities are already in the text. Taken together, the two comments indicate to us that Figure 1 is not adding anything (in fact may be confusing) and we have deleted it.

Discussion

Ln 355 Please explain how you obtained the estimate that an additional 9-12% of prescriptions can be turned into delayed prescriptions?

We have expanded the description of our approach in the Discussion (paragraph 2). The estimation and assumptions are described fully in the Supporting Information.

Ln 416 regarding parents starting from a low knowledge basis: see my earlier comment on the risk for biased population in this study, towards more educated persons/parents from higher socio-economical classes. In view of the current economical and societal changes, it will be important to retrieve data from other, more vulnerable/hard to reach groups in our societies as well

As with the earlier comment, we agree and have added this point to the Discussion (paragraph 12)

**Editor**

Please supply a non-author contact for inquiries about access to study data, as required by PLOS' data policy.

The data availability statement has been updated to:

Data cannot be shared publicly because the terms of our ethics clearance included that the data would be held on secure networks at our University. Data are available from the Information Governance Committee, Nuffield Department of Population Health, University of Oxford (contact via ig.support@ndph.ox.ac.uk) for researchers who meet the criteria for access to confidential data.

Please adapt the title to better match journal style. We suggest "Public preferences for delayed or immediate antibiotic prescriptions in UK primary care: a discrete choice experiment".

We have changed the title to reflect this style. However we have omitted the word ‘discrete’, as referring to the study as a ‘DCE’ will lead readers to expect a design with multiple profiles in each choice question. As discussed in our response to the reviewer above, our study differs from this format.

Please quote the dates over which the study was done in the abstract.

Done.

In the abstract and elsewhere in the paper, please add p values alongside 95% CI, where available.

P values have been added to the 95% CIs throughout the choice models.

In the final sentence of the “methods and findings” subsection of your abstract, summarizing the study’s main limitations, we ask you to add 1-2 further limitations (e.g., the issue of unclear relevance of your findings to other populations).

Further limitations have been added; the focus on immediate vs delayed prescription, and the issue of generalisability to population groups under-represented in online surveys.

After the abstract, we will need to ask you to add a new and accessible "author summary" section in non-identical style. You may find it helpful to consult one or two recent research papers published in PLOS Medicine to get a sense of the preferred style.

Author summary has been added.

Early in the methods section, please state whether the study had a protocol or prespecified analysis plan, and if so attach the document(s) as a supplementary file (referred to in the methods section). Please highlight any analyses that were not prespecified.

We did not have a specific protocol or analysis plan. The use of the mixed-effect logistic regression model, and the respondent characteristics evaluated, were pre-determined, and were built in to the experimental design and the questions asked in the survey. We expected to examine interactions between respondent characteristics and the attributes in order to explain heterogeneity of responses, but had not pre-specified which attributes or characteristics would be studied; these were guided by the data. We have noted that the interaction attributes were not pre-specified in the Methods, under ‘Analysis’, paragraph 3.

At line 332, please soften the wording slightly to: "Our findings suggest that the most important factors ...".

Changed.

Throughout the paper, please quote p values as "p<0.001" or exact values.

Done.

Throughout the paper, please provide greater emphasis on reported observations, e.g., at line 203 "... of the parent sample reported that they were employed or self-employed ...".

Changed - several places in Results, under ‘Respondent Characteristics’, paragraphs 2-3, and one in the Discussion, paragraph 7.

Please substitute "sex" for "gender" as appropriate throughout the text.

Done (8 cases).

Throughout the article, please adapt reference call-outs to the following style: "... Europe [6,7,12].".

Done.

In your reference list, please ensure that journal names are abbreviated consistently, e.g., "Cochrane Database Syst Rev." for reference 4.

Done – for example Cochrane, BMJ Open, Journal of Antimicrobial Chemotherapy, are now in consistent format.

Please add a completed checklist for the most appropriate reporting guideline, which we suspect will be STROBE, referred to in the methods section. In the checklist, please refer to individual items by section (e.g., "Methods") and paragraph number rather than by page or line numbers, as the latter generally change in the event of publication.

We attach a completed checklist as developed by the International Society for Pharmacoeconomics and Outcomes Research (ISPOR) for conjoint experiments. We believe this is the most appropriate checklist, as it was developed specifically for multi-attribute choice experiments in health care, where the implicit value of an attribute is derived from an overall score for a profile. The checklist aims to provide guidance on good research practice, and some elements of the checklist describe methodological considerations rather than requirements. As a result of completing the checklist, we have made minor adjustments to the Methods section to ensure the checklist items are explicitly described.

Authors’ note to Editor:

Following further discussions with the team, and comments from the reviewers, we have reconsidered our estimate of the number of antibiotic prescriptions that could be avoided (Discussion, and Supporting Material). Having considered additional factors that could affect this estimate (described in Supporting Material, Section 5), we have softened our estimate of the number of prescriptions to ‘conservatively, over half a million’ (Discussion, paragraph 2) which reflects the sensitivities we have examined, and avoids presenting an overly specific number. We trust this revision will be acceptable.
