# Supplementary material for: Public preferences for delayed or immediate antibiotic prescriptions in UK primary care: A choice experiment
Source: PLoS Med. 2021 Aug 30;18(8):e1003737. doi: 10.1371/journal.pmed.1003737 (PMC8439451; doi:10.1371/journal.pmed.1003737)
Supplement: Attachment — Submitted filename: response to editor and reviewers.docx [file pmed.1003737.s012.docx]

**PMEDICINE-D-19-04469R1**

**RESPONSES TO EDITORIAL REQUESTS AND REVIEWER FEEDBACK**

**Editorial requests:**

Please make that "has been shown ..." in the second line of the abstract.

Done (p2 in the tracked-changes version).

Please quote summary details on respondent demographics in your abstract, and the survey response rates.

Sentence added to second paragraph of Methods and Findings section (p2):

‘The survey was completed by 802 adults and 801 parents (75% of those who opened the survey). The samples reflected the UK population in age, sex, ethnicity and country of residence’.

In the abstract and throughout the text, please quote p values alongside 95% CI, where available.

Done (pp2-3, p22, p23).

In your abstract, please quote quantitative data, including 95% CIs and p values, for the following results: “Respondents were less likely to choose delayed prescription with increasing duration of illness. Probabilities were similar for parents considering treatment for a child. However, parents differed from the adult sample in giving higher weighting to the duration of illness, and lower weighting to future disruption of usual activities. Females were more likely to choose a delayed prescription than males, and were particularly likely to do so for minor cough symptoms.”

We have added odds ratios or probabilities as appropriate, along with 95% CIs and p-values. This required a minor adjustment to the observation about sex, to make the sentence clearer once the quantitative date were inserted (pp2-3).

Please condense the discussion of study limitations in your abstract to a single sentence, concluding the "Methods and findings" subsection, e.g., "Study limitations include the hypothetical nature of the choices offered, which may not reflect real-life behaviour; the absence of an option of no prescription; and the possibility that study respondents may not be representative of the population as a whole or of groups within it.".

We have adopted much of your suggested concise version; we felt it was important to retain a reference specifically to the possible effect of online sampling (p3).

Please adapt the first sentence of the "Conclusion" subsection of our abstract to begin "In this study, we found that delayed prescription ..." or similar.

Done (p3).

Please make that "... (lack of) a role ..." in the author summary.

Done (p4).

Please condense the discussion of the study aim and design at the end of the Introduction section.

We have condensed this discussion by deleting text, restructuring, and moving some detail to the Methods section. (pp5-6). We hope this is now sufficiently concise.

As discussed in your cover letter, please state explicitly early in your methods section that the study did not have a prespecified analysis plan.

Statement added to the Analysis section of the Methods (p13):

‘Use of the mixed-effects logistic regression model, and the respondent characteristics to evaluate, were pre-determined; further analyses were not pre-specified.’

In your methods section, please add a brief discussion on the question of informed consent for study respondents.

Statement added to the Data Collection section of the Methods (p12):

‘Respondents were provided with on-screen information about the study, and gave informed consent to participate by clicking a ‘Yes, I agree to take part’ button.’

In the discussion section, where you mention that you "quantify the trade-offs", please amend this to "attempt to quantify" or "estimate the trade-offs", or similar.

We have amended to: ‘To our knowledge, this is the first study in the UK to attempt to quantify the trade-offs…’ (p25).

Please remove information on funding from the Acknowledgements section at the end of your main text. This information will appear in the article metadata, via entries in the submission form.

Done (p31).

Please ensure that all references meet journal format. For example, "BMJ" will suffice as the journal name for reference 4, and this citation may be lacking some access information.

Please ensure that journal names are abbreviated consistently.

We have checked reference abbreviations (including BMJ, J Antimicrob Chemother, Br J Gen Pract) to bring them in line with NCBI abbreviations; we also checked journal references have doi’s for ease of access (pp31-33).

Please check that the URL provided for reference 45 functions, and add accessed dates to the web references.

We have updated the link for reference 45, and added Accessed dates to all web page references.

**NOTE TO EDITOR:**

On checking the manuscript, we noticed that in a previous edit, one of the quota terms (ethnicity) had been accidentally deleted from the Methods, Data Collection (p12). We have now corrected this deletion.

**Comments from Reviewers:**

*** Reviewer #1:

The authors have addressed all my points

Thank you for re-reviewing our paper.

*** Reviewer #4:

Thank you for the opportunity to contribute to the review for this article, which I found to be well-written and describing an interesting and important topic. Thoughtful and thorough comments have already been provided by three reviewers, and in my opinion the authors have addressed the points raised and the article is suitable for publication in PLoS Medicine.

Thank you for reviewing and recommending the suitability of our article for PLoS Medicine.

I have the following minor points:

- Agreed that a blocked design would have been interesting to better explore the space and potentially better opportunities for interactions - this seems worth mentioning as a limitation but not a "fatal flaw"

We agree that a blocked design would have given us additional information, which we address as a limitation (p29), and also that it does not undermine the research.

- Multiple reviewers pointed out the absence of a 'no prescription' option and the authors address this in the discussion. However, I would argue that in this context, there may not be need for such an option - presumably implicit in the delayed prescription is the potential for the patient (or child)'s condition to improve spontaneously and allow them to spare the antibiotics. In that case, it would seem that a delayed prescription already carries the potential for 'no medication' which would be functionally equivalent to 'no prescription', but leaves this decision and its timing to the patient and in that case could be seen as a more empowering/patient-centric approach than an immediate prescription

We agree that delayed prescription contains an implicit ‘no prescription’ option if the patient’s condition improves in the way that the GP would explain. We have added a sentence to that effect in the discussion of this issue (p 29).

- Reviewers noted the scale of the variables for reporting odds ratios - of note, one option might be to report values for, e.g. 5% or 5 minutes etc., to more naturally track with the differences between attribute levels. Alternatively (or in addition to), I have contributed to DCE studies in the past where we have used barplots to communicate the probability of making a particular selection across several representative collections of attributes and have had good feedback in that as clarifying interpretation of the regression results.

Thank you for this suggestion on reporting the regression models. We did consider scaling the odds ratios other than for a unit change in the attribute; indeed, this approach has been useful in other DCEs for presenting bar charts of coefficients that would otherwise be difficult to see, as well as tracking with the numbers provided to respondents. However in the table, we have retained the unit change scaling, as the approach that readers are perhaps most used to, and (perhaps intuitively for readers) where the odds ratios closer to unity are also the ones with higher p-values.

We agree that probabilities based on scenarios are a helpful way of illustrating the interpretation of regression results. We chose to do so by focusing on the ‘symptoms’ attribute, which has a large effect on the probabilities, and also allowed us to illustrate the effect of the interaction with respondent characteristics such as sex. We then took one sore throat scenario and used it as the basis for our estimate of potential for reduction in antibiotic consumption. However in the interests of the length of the article, we have chosen not to also put a set of scenarios in the paper.

*** Reviewer #5:

Many thanks for the opportunity to review this revised manuscript. The study represents a valuable addition to the literature and advances our knowledge of preferences for delayed versus immediate antibiotic prescriptions. The authors have responded to the peer-review comments and have amended the manuscript appropriately. The theoretic reasoning for immediate versus delayed, and the absence of a "no prescription" option - is adequately described, with appropriate discussion around the limitations of this design. The experiment is now clearly described. The use of the term 'delayed' and use of 'back-up' in parentheses, or lay versions - was justified and meets with the understanding of a wide ranging readership. The added clarity in the manuscript allows readers to interpret the findings based on the clearly described hypothetical scenario, limitations are acknowledged and interpretation is supported by the transparency in reporting. The manuscript appears to have undergone appropriate revision to address the reviewers comments, and improving the clarity of a valuable study.

Thank you for reviewing our revised manuscript and evaluating our response.
